# An Update on the Role of Lymphatic Function in Skin Inflammatory Disorders: A Scoping Review

**DOI:** 10.7759/cureus.77981

**Published:** 2025-01-25

**Authors:** Irina Ter-Ovanesyan, Michelle Tashjian, Stephanie Escruceria, Richard Fernandez, Bryant Estadella, Harvey N Mayrovitz

**Affiliations:** 1 Immunology, Nova Southeastern University Dr. Kiran C. Patel College of Osteopathic Medicine, Fort Lauderdale, USA; 2 Medical Education, Nova Southeastern University Dr. Kiran C. Patel College of Allopathic Medicine, Davie, USA

**Keywords:** autoimmune, cytokines, inflammation, lymphatics, lymphedema, skin, skin cancer, skin health, skin infection, wound healing

## Abstract

The lymphatic system is essential in maintaining skin health through coordinated immunological actions. This review explores the relationship between lymphatic function and skin health, as well as the impact of lymphatic dysfunction in the development and progression of inflammatory skin disorders. A systemic search was conducted in the Web of Science, Embase, and Ovid MEDLINE databases, following the Preferred Reporting Items for Systematic Reviews and Meta-Analyses Extension for Scoping Reviews (PRISMA-ScR) guidelines. Included studies were peer-reviewed human or animal research published in English from 2014 to 2024, focusing on inflammatory skin disorders, including skin cancer, autoimmune skin diseases, and infectious skin diseases. A total of 1232 citations were identified, with 37 studies meeting the eligibility criteria after assessment and critical appraisal.

The review’s findings highlight the essential role of lymphatics in maintaining skin health, mitigating inflammatory, infectious, and skin cancer-related processes, and delaying the effects of skin aging. The mechanisms underlying lymphatic function in these processes are complex, with some aspects needing further investigation. However, the evidence indicates that a well-functioning skin lymphatic system, supported by various cytokines, aids in reducing the inflammatory state, reduces inflammation, alleviates lymphedema, and prevents lymphatic stasis, which can increase infection risk. Several studies demonstrated that restoring lymphatic function through improved neutrophil migration and cytokine responses reduces the spread of infectious diseases.

## Introduction and background

The lymphatic system is essential in maintaining skin health through coordinated immunological actions. Disruption of normal lymphatic function and the resulting inflammation are associated with various skin conditions, including cancer [1], infectious diseases [2], and autoimmune disorders [3]. The inflammatory response in these conditions is linked to the upregulation of the vascular endothelial growth factor (VEGF) signaling pathway, particularly VEGF-A [4]. Notably, VEGF-C has been shown to reduce chronic inflammation and improve lymphatic function [5,6]. In addition, the lymphatic system helps maintain skin homeostasis by draining toxins and bacteria [7] and directing antigen-presenting cells to lymph nodes, playing an important role in the immune responses against pathogens [8].

Impairment of the processes described above can lead to lymph fluid stasis, contributing to the development of inflammation. Chronic inflammation has been shown to stimulate both angiogenesis and lymphangiogenesis, processes that are closely studied due to their role in promoting cancer metastasis [9]. Angiogenesis is primarily regulated by the VEGF-A signaling pathway, where pro-angiogenic factors activate and proliferate endothelial cells. In psoriatic lesions, angiogenesis is characterized by significant vasodilation, increased vascular permeability, and blood vessel elongation [10]. VEGF-induced angiogenesis in psoriasis may contribute to the pathogenesis and progression of the autoimmune condition [7]. This review explores the specific role of lymphatic function in maintaining skin health and the effects of its disruption in various inflammatory skin disorders. In addition, it examines VEGF-A and VEGF-C signaling pathways as potential targets for suppressing inflammation and enhancing lymphatic function. Despite the growing recognition of the lymphatic system's importance in skin health and inflammation, comprehensive reviews on this subject are lacking. A preliminary search of databases such as MEDLINE, the Cochrane Database of Systematic Reviews, and JBI Evidence Synthesis found no ongoing or published systematic or scoping reviews on this topic. Therefore, this scoping review aims to assess the extent of the literature on the role of lymphatic function and dysfunction in skin health and inflammatory skin disorders.

## Review

Materials and methods

*Search Strategy* 

This scoping review searched for primary peer-reviewed studies published in English from 2014 to 2024, using three databases: Embase, Ovid MEDLINE, and Web of Science. Eligible studies had to address skin cancer, autoimmune skin conditions, or skin infections in relation to lymphatic function. Studies that did not discuss or measure lymphatic function were excluded. 

Search Terms

The following search terms and phrases were used: “skin disease” or “autoimmune skin disease” or “bullous skin disease” or “dermatitis” or “erythematosquamous skin disease” or “skin infection” or “skin edema” or “skin tumor” or “benign skin tumor” or “skin cancer” or “diabetic skin disorder” or “scalp pruritus” or “Skin disease*” or “dermatitis” or “skin infection*” or “skin edema” or “skin tumor*” or “skin cancer*” or “skin disorder*” or “scalp pruritus” or “inflammatory skin condition*” or “psoriasis” or “atopic dermatitis” or “melanoma” AND “Lymphangiogenesis” or “lymphatic system” or “lymph* function” or “lymph* dysfunction” or “lymphangiogenesis” or “lymphangio*” or “lymph* drainage” or “lymph* vessel*” or “lymph* system” or “VEGF-A” or “VEGF-C” or “vasculotropin A” or “vasculotropin c”. These keywords were combined in various ways to broaden the search.

Search Outcomes

**Figure 1:**
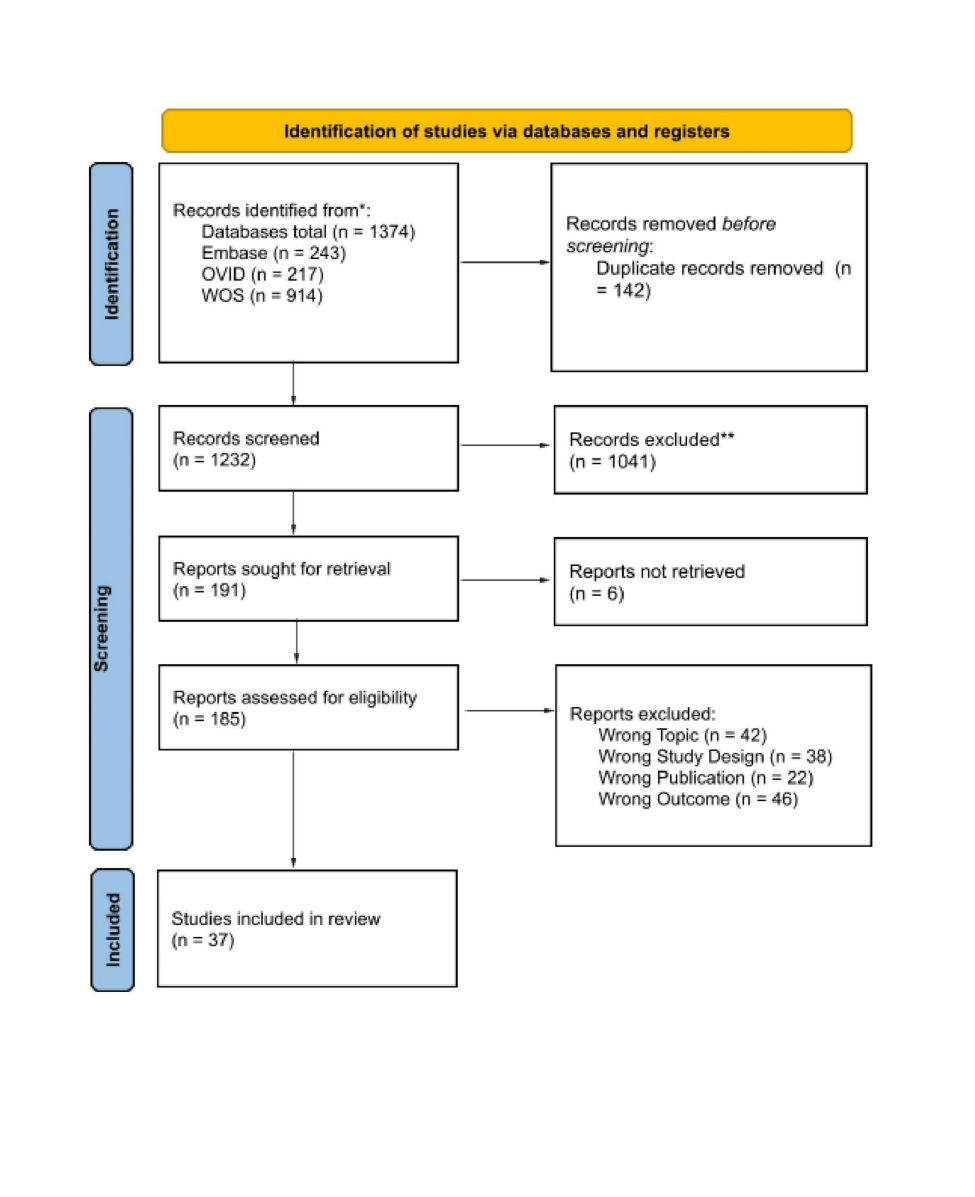
Search approach The diagram shows the Preferred Reporting Items for Systematic Reviews and Meta-Analyses (PRISMA) flowchart, which quantitatively depicts the study selection process and reasons for excluded reports.

The initial search identified 1374 citations. After removing 142 duplicates, 1232 studies remained for screening. Titles and abstracts were reviewed, and a consensus was reached to include 185 articles for further consideration. Articles were excluded based on study type and other factors. The screening and selection process is summarized in the Preferred Reporting Items for Systematic Reviews and Meta-Analyses (PRISMA) flowchart (Figure 1). The Joanna Briggs Institute Appraisal Tools were used to assess the methodological quality, possible biases, and relevance of the included studies [11]. After the quality appraisal, 42 studies were excluded due to the wrong topic, 38 due to incorrect study design, 22 due to incorrect publication type, and 46 due to irrelevant outcomes. This left 37 articles for the final scoping review.

Synthesis of Results

First, we explore skin health, emphasizing the role of lymphatics in protecting against pathogens, promoting wound healing, exerting anti-inflammatory effects, supporting metabolism, and mitigating aging. The studies were categorized based on disease type, specifically cancer, autoimmune skin disorders, and infectious skin disorders. We then review promising therapeutic approaches that target lymphatic function to treat these conditions.

Healthy skin and lymphatics

While the role of blood vessels in skin health has been extensively studied, the skin lymphatic system has received less attention and is not as well understood. Past research suggests that “lymphatic vessels are crucial for immune surveillance, as they serve as main transport routes for cells and inflammatory mediators to lymph nodes, where immune responses are mounted” [12]. Inflammation causes blood vessels to swell and leak inflammatory factors, leading to edema. In response to increased interstitial fluid pressure, lymphatics dilate, allowing fluid and inflammatory cells to enter the lymphatic vasculature and be removed from the inflamed tissue. VEGF-3 and VEGF-C are key players in this process: VEGF-3 promotes lymphatic expansion to combat inflammation, while VEGF-C stimulates endothelial cell proliferation and migration. VEGF-A induces lymphangiogenesis, the growth and formation of new lymphatic vessels, which occurs in both normal tissue development and pathological processes such as inflammation, wound healing, lymphedema, and cancer [13]. Together, these factors contribute to lymphatic function, helping maintain healthy skin and the ability to fight inflammation. Furthermore, skin lymphatics assist in transporting cutaneous high-density lipoprotein (HDL) from the skin to the liver for degradation. Impaired lymphatic function, partially the lack of SR-B1 receptor on lymphatic capillaries, can decrease HDL transit out of the skin [14]. This suggests that healthy skin, supported by the lymphatic system, plays a role in balancing body metabolism. In addition, lymphatics are essential for wound healing and delaying tissue fibrosis, which can slow the aging process. Decreased VEGF-3 signaling contributes to skin aging by causing lymphatic endothelial cell apoptosis, reduced lymphatic pumping and migration, and decreased production of chemokines and adhesion molecules [15]. Therefore, proper VEGF-3 and VEGF-C function is vital for maintaining healthy skin through effective wound healing, inflammatory clearance, metabolic maintenance, and prevention of premature skin aging.

Skin inflammation

While inflammation and lymphatic function have generally been reviewed in various contexts, the specific role of lymphatic function in the progression (or lack thereof) of skin inflammatory disorders remains underexplored [12]. The relationship between lymphatic vasculature function and cutaneous health has been previously investigated, particularly in the context of tumor-associated lymphangiogenesis. Inflammatory processes have been shown to influence the progression of cutaneous malignancies, depending on the inflammatory environment [13]. To replicate these conditions, several murine models have been used. In tumorigenesis, murine models were infected with k-cyclin, a Kaposi sarcoma-associated herpesvirus latent-cycle gene, to demonstrate a severe lymphatic dysfunctional state. These models were then subjected to a two-stage carcinogenesis protocol and imiquimod-induced psoriatic lesions. Tumor progression was accelerated in these lymphedematous murine models, with exacerbated skin inflammation and elevated pro-inflammatory cytokine levels compared to the wild type [16]. In addition, some diseases involve the progressive aberrant development or obliteration of lymphatic vessels based on the severity and duration of the skin disease. For example, systemic sclerosis exhibits progressive lymphangiopathy where the loss of cutaneous lymphatic vessels correlates with the disease progression [17]. Cutaneous delayed-type hypersensitivity reactions have been studied in murine models overexpressing VEGF-A, showing multiple phenotypic similarities akin to human psoriasis, implying that VEGF-A is an important factor in the pathogenesis of psoriasis [18].

In another study involving psoriasis models, researchers targeted VEGF, a key factor in lymphatic function, to assess its effect on inflammation [19]. Inhibition of VEGF reduced inflammation in psoriasis, suggesting therapeutic potential for skin disorders. Another study noted an increase in VEGF-A, a protein of the VEGF family, which has been shown to exacerbate psoriasis plaques by promoting angiogenesis [20], leading to leaky vessels and dysfunctional lymphatic drainage. In obese mouse models with psoriasis and atopic dermatitis, administration of VEGF-C demonstrated a marked reduction in skin inflammation [21]. This highlights how compromised lymphatic function can amplify these inflammatory skin responses. A study on UVB-induced skin inflammation found increased lymphatic vessel expansion and decreased VEGF-C levels, which led to edema and lymphatic dysfunction [22]. Introducing VEGFR-3, which binds VEGF-C, stimulated lymphatic endothelial cell proliferation and promoted lymphangiogenesis in mouse models, alleviating UVB-induced skin inflammation. In another study, UVB exposure resulted in VEGF-A overexpression, which worsened skin damage [23]. There is potential to counteract UVB-induced inflammation by blocking VEGF-A. In mouse models, UVB and VEGF-A disruptions to lymphatic drainage can potentially be alleviated through increased VEGF-C administration. While VEGF remains an important player, a study highlights the role of lymphatic endothelial cells (LECs) in acute cutaneous hypersensitivity, noting that LECs affect lymphatic function through several vessel leakages and reduced lymph ejection, independent of VEGF [24].

Skin cancer and lymphatics

De Moura et al. found that CD147 expression aids with melanoma-associated lymphangiogenesis by upregulating the PROX-1 transcription factor, podoplanin, and VEGFR-3 in melanoma lymph nodes [25]. In addition, CD147 facilitates tumor cell dissemination through the lymphatic system by stimulating lymph endothelial cells to proliferate, migrate, and survive in tubulogenesis, a process essential for endothelial maturation and optimal functioning [25]. Lymphatic remodeling was further explored by de Jel et al., who studied CYLD expression in melanoma tissues. CYLD-deficient mice showed increased lymphatic vessel growth, promoting lymphangiogenesis and a more favorable microtumor environment [26]. Furthermore, extracellular vesicles in regional lymph nodes of patients with primary cutaneous melanoma also function as traffickers in the lymphatic vasculature, contributing to the formation of the first pre-metastatic niche [27,28]. Throughout the literature, it has been noted that the vascular endothelial growth factor receptor-1 (VEGFR-1) is frequently expressed in melanoma, and its activation by VEGF-A promotes tumor migration and invasiveness [29]. Atzori et al. investigated the role of VEGFR-1 in resistance to vemurafenib (a BRAF inhibitor), finding that melanoma cells expressing VEGFR-1 were more invasive than VEGFR-1-deficient cells, suggesting VEGFR-1’s involvement in metastatic tumor growth [29]. 

While lymphangiogenesis in melanoma has long been linked to metastasis and poor prognosis, Sasso et al. investigated it in a different context [30]. The growth of lymphatic vessels was shown to enhance cancer immunotherapy and boost T-cell immunity [30]. In their study, a vaccine was formulated with the pro-lymphangiogenic growth factor VEGF-C and tested in mouse melanoma models. It was reported that the VEGF-C vaccine resulted in a higher frequency of antigen-specific T cells, providing effective tumor control and long-term immunity [30]. Yeh et al. also identified VEGF-C as a potential biomarker for tumor metastasis and a therapeutic target in skin cancer treatment [31]. Lymphangiogenesis is often found in areas with stromal inflammatory cells in melanoma, further supporting VEGF-C’s role in the lymphatic response to cancer growth [32].

Furthermore, Yoon et al. highlighted the importance of developmentally regulated GTP-binding protein 2 (DRG2) in regulating VEGF-A expression in melanoma cells [33]. Knockdown of DRG2 significantly reduced VEGF-A in B16F10 cells and impaired endothelial tube formation, inhibiting cancer spread. However, high DRG2 expression was correlated with a lower survival rate in melanoma patients [33].

Autoimmune skin disorders and lymphatics

Chronic Localized Fibrosing Leukocytoclastic Vasculitis

A case study of chronic localized fibrosing leukocytoclastic vasculitis (CLFLCV) evaluated at the histopathological level revealed a high presence of eosinophils and macrophages localized to areas of fibrosis. This finding suggests a cycle of vasculitis progressing to the accumulation of layers of fibrous tissue [34]. The pathophysiology is thought to be related to chronic lymphedema, where persistent lymphostasis and inflammation result in permanent tissue overgrowth. In a separate case study of intralymphatic histiocytosis in Morbihan disease, dilated lymphatic vessels and interstitial dermal edema were noted to contribute to inflammation and disease progression [35]. Further studies are needed to establish a definitive causal relationship. 

Psoriasis

Korenfeld et al. found that CD5+ dendritic cells derived from dermal progenitor cells express high levels of TNF-alpha and LT-alpha/beta, driving the autoimmune response in psoriasis [36]. Furthermore, VEGF-C was shown to activate lymphatics, promoting the resolution of inflammation in mice with psoriasis-like skin. Targeted deletion of endothelial IL-7 receptor in mice reduced lymphatic drainage and increased edema in psoriasis-like skin, suggesting another contributing factor to impaired lymphatic function in skin disease manifestation [37].

Infectious skin disorders and lymphatics

Olszewski et al. studied the infectious complication of lymphedema, known as dermato-lymphangio adenitis, which is caused by *Staphylococcus epidermidis* and *aureus* from the patient’s natural skin flora in the setting of lymphatic dysfunction [38]. They found that individuals with a genetic polymorphism of TNF-alpha are more prone to lymphatic dysfunction, leading to lower limb lymphedema and a higher risk of developing dermato-lymphangio adenitis. Vallhov et al. demonstrated that Malassezia’s extracellular vesicles interact with host macrophages and the ICAM-1 ligand on keratinocytes [39]. This interaction suggests a mechanism for fungal infection spread and presents a potential therapeutic target to halt infection progression. In addition, the optimal functioning of skin lymphatics is necessary for rapid neutrophil recruitment to lymph nodes in order to prevent the systemic spread of bacteria [40]. Dysfunctional lymphatic vessels impair the transport of bacterial antigens and infection-induced regulatory factors to lymph nodes, increasing the risk of disseminated infection.

Therapeutic approaches

Olmeda et al. discussed the potential of imaging the lymphatic vasculature during tumor progression as a strategy to develop therapeutic approaches for metastatic melanoma [41]. Lymphoscintigraphy, a common imaging method used in the clinical setting, helps identify tumor-draining lymph nodes, guiding medical interventions [42]. A mouse model study showed that Oleuropein inhibits tumor growth and lymph node metastasis while also suppressing angiogenesis and lymphangiogenesis [42], suggesting a potential approach to suppress tumor progression through skin lymphatics, though further human studies are needed. 

Wu et al. tested the effectiveness and mechanisms of secukinumab in treating psoriasis [43,44]. Their findings demonstrate that secukinumab effectively cleared psoriasis skin lesions by reducing the expression levels of several lymphatic cytokines involved in the inflammatory cascade response associated with IL-17A. IL-17A is linked to prolonged disease duration and increased inflammatory cytokine production in psoriasis. These results highlight the potential of targeting IL-17A within skin lymphatics to treat psoriasis. Another promising treatment, gentiopicroside, exhibited antiproliferative, apoptosis-promoting, anti-inflammatory, and antiangiogenic effects in mice, suggesting its potential for psoriasis treatment [45]. Further research can develop novel treatments that not only address lymphatic disorders but also chronic inflammatory disorders. Although these therapies are promising, further research in human subjects is needed to fully explore the role of lymphatics in various inflammatory skin conditions.

Discussion

The purpose of this review was to explore the various roles of the lymphatic system in skin health and skin inflammation and identify potential therapeutic targets for lymphatic dysfunction in inflammatory skin disorders. The findings highlight the critical role of the lymphatic system in maintaining skin health and its involvement in the pathology of inflammatory skin conditions such as cancer, autoimmune diseases, and infections. Lymphatic dysfunction emerges as a common factor linking various skin conditions, including melanoma, psoriasis, and infectious skin diseases, highlighting the complex interplay between lymphatic function and skin pathologies. However, while associations exist, they are not universally confirmed across all mentioned conditions, especially in the absence of robust human clinical studies. The association of chronic inflammation with lymphatic dysfunction, as observed in metastatic melanoma and psoriasis, highlights the lymphatic system's fundamental role in maintaining dermal health [25,26]. CD147 overexpression in cancer cells catalyzes lymphangiogenesis and provides a nuanced understanding of the lymphatic system's involvement in cancer progression [29]. Moreover, the distinct roles of VEGF-A and VEGF-C in lymphatic biology reveal the intricate interplay within skin pathology: VEGF-A exacerbates inflammatory conditions through angiogenesis [31], whereas VEGF-C represents a viable target for ameliorating inflammation and enhancing lymphatic functionality [22].

Study limitations 

Language

A potential limitation of this review is the exclusion of non-English language studies. Relevant research published in other languages may have been overlooked, and the extent to which such studies could have contributed to the topic remains unknown due to the English-only search filter.

Experimental Animal Models 

Several studies in this scoping review utilize murine models to investigate various pathologies and their associated immunological factors. While these models provide valuable insight into immune responses and disease mechanisms, the applicability of these findings to other animal models or human conditions warrants further consideration. The reliance on murine research may not fully capture the complexity of lymphatic function across different species or accurately reflect the nuances of human dermatological health and disease.

## Conclusions

The review highlights the crucial role of lymphatics in maintaining healthy skin, mitigating skin inflammation, infections, and skin cancer-related processes, and delaying skin aging. While the specific mechanisms of lymphatic involvement are complex and warrant further research, the findings emphasize that a healthy lymphatic system, supported by various cytokines, helps reduce inflammation and lymphedema, preventing lymphatic stasis and minimizing the risk of infection. Several studies have shown that restoring lymphatic function reduces the dissemination of infectious diseases by enhancing neutrophil migration and cytokine responses.
